# Short-Term Therapeutic Adherence of Hospitalized Older Patients with Oropharyngeal Dysphagia after an Education Intervention: Analysis of Compliance Rates, Risk Factors and Associated Complications

**DOI:** 10.3390/nu14030413

**Published:** 2022-01-18

**Authors:** Jesús Mateos-Nozal, Elisabeth Sánchez García, Beatriz Montero-Errasquín, Estela Romero Rodríguez, Alfonso J. Cruz-Jentoft

**Affiliations:** Servicio de Geriatría, Hospital Universitario Ramón y Cajal (IRYCIS), 28036 Madrid, Spain; elisabet_sanchez@hotmail.com (E.S.G.); beatriz.montero@salud.madrid.org (B.M.-E.); estelina_3@hotmail.com (E.R.R.); alfonsojose.cruz@salud.madrid.org (A.J.C.-J.)

**Keywords:** deglutition disorders, aged, treatment adherence and compliance, complications, mortality

## Abstract

Oropharyngeal dysphagia (OD) is associated with adverse outcomes that require a multidisciplinary approach with different strategies. Our aim was to assess the adherence of older patients to dysphagia management recommendations during hospitalization, after a specific nurse guided dysphagia education intervention and to identify short term complications of OD and their relationship with short-term adherence. We carried out a prospective observational study in an acute and an orthogeriatric unit of a university hospital over ten months with a one-month follow-up. Four hundred and forty-seven patients (mean age 92 years, 70.7% women) were diagnosed with dysphagia using Volume-Viscosity Swallow Test (V-VST). Compensatory measures and individualized recommendations were explained in detail by trained nurse. Therapeutic adherence was directly observed during hospital admission, after an education intervention, and self-reported after one-month. We also recorded the following reported complications at one month, including respiratory infection, use of antibiotics, weight loss, transfers to the emergency department, or hospitalization). Postural measures and liquid volume were advised to all patients, followed by modified texture food (95.5%), fluid thickeners (32.7%), and delivery method (12.5%). The in-hospital compliance rate with all recommendations was 37.1% and one-month after hospital discharge was 76.4%. Both compliance rates were interrelated and were lower in patients with dementia, malnutrition, and safety signs. Higher compliance rates were observed for sitting feeding and food texture, and an increase in adherence after discharge in the liquid volume and use of thickeners. Multivariate logistic regression analysis showed that adherence to recommendations during the month after discharge was associated with lower short-term mortality and complications (i.e., respiratory infection, use of antibiotics, weight loss, transfers to the emergency department, or hospitalization). One-third of our participants followed recommendations during hospitalization and three-quarters one month after admission, with higher compliance for posture and food texture. Compliance should be routinely assessed and fostered in older patients with dysphagia.

## 1. Introduction

Oropharyngeal dysphagia (OD) is common in hospitalized older patients with prevalence rates between 7.9% and 86% [1,2,3]. This heterogeneity is attributed to the inclusion of different populations and various diagnostic tools. OD is associated with adverse outcomes such as respiratory infections, malnutrition, dehydration, hospital readmissions, institutionalization, costs, and mortality [4,5,6,7].

OD clearly impacts on the nutritional status of the older patient, being connected with malnutrition bidirectionally (higher risk of OD in malnourished patients and higher risk of malnutrition in patients with OD and inflammation), with dehydration (a classical complication of OD patients), and with anorexia, frailty, and sarcopenia (higher risk of these syndromes among OD patients). Older patients with OD usually present impaired health status with periodontitis, caries or edentulism that is also related to nutritional status [4,5].

A multidisciplinary approach is required to manage OD in older persons, and its treatment should include rehabilitation and compensatory techniques, as proposed by different societies and authors [4,8,9,10,11]. These authors and also the European Society for Clinical Nutrition and Metabolism recommended the use of textured-modified for patients with OD [12]. However, this recommendation is graded as good clinical practice (GCP) due to the absence of literature assessing this topic [13]. Swallow rehabilitation and maneuvers are also advised for older patients [4,8,9,10,11] and patients with different neurological conditions [14]. The European Dysphagia Working Group also stated the importance of compliance with treatment [4].

A standard definition of compliance is when a patient’s behaviors coincide with healthcare providers’ recommendations for health and medical advice, including medications, diets, and lifestyle changes. In contrast, therapeutic non-compliance occurs when a patient’s behavior lacks congruence with the healthcare provider´s recommendation [15]. The compliance rate for short-term therapy was higher (70–80%) than for long term medication (40–50%) and lifestyle changes (20–30%) [16]. 

Compliance with OD management recommendations is also low, from 21.9 to 51.9% in a review of 12 studies with OD [17]. Most of the studies identified in this review focused on head and neck cancer patients and their swallowing rehabilitation. Only three studies included different etiologies and evaluated adherence to diet modification [17]. Another recent study observed poor therapeutic compliance to fluid thickening and texture modification in older patients with dementia and OD after hospitalization [18].

Therefore, the aim of this study was to assess the adherence of older patients to recommendations (food and liquid textures, liquid volume, posture and delivery method), after an individualized educational intervention, during hospitalization and one month after discharge to OD individualized management recommendations. A secondary objective was to identify short-term complications of OD and their relationship with short-term treatment adherence.

## 2. Materials and Methods

### 2.1. Study Design

**Figure 1:**
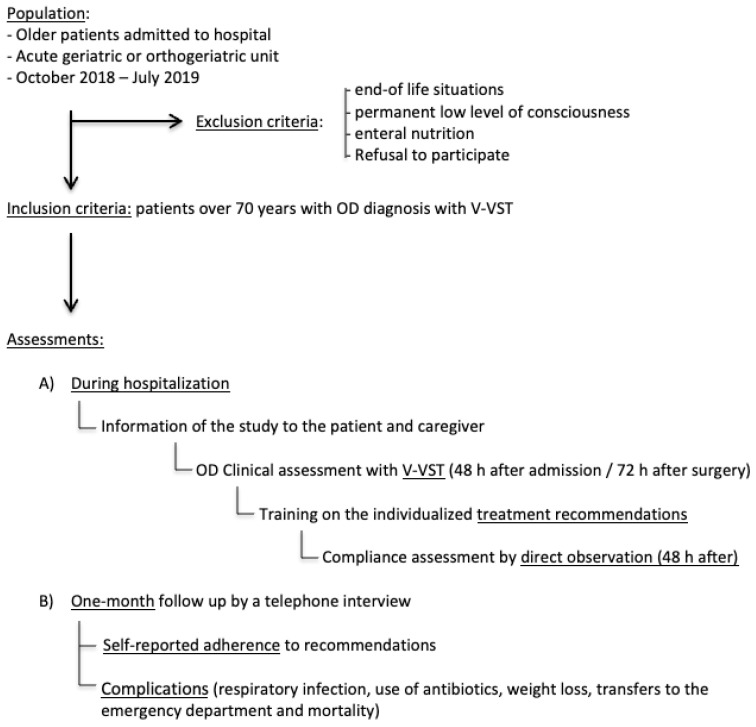
Study design and assessments. Abbreviations: OD: Oropharyngeal Dysphagia, V-VST: Volume-Viscosity Swallow Test, h: hour.

This was a prospective, observational, pragmatic study on patients consecutively admitted to an acute geriatric unit or an orthogeriatric unit of a university hospital over ten months, with one-month follow up, to describe the therapeutic adherence of older patients with OD. During admission, patients received OD management recommendations according to clinical dysphagia assessment. The study included at least two visits during hospitalization and a telephone assessment one month after discharge, as it is presented in Figure 1. 

### 2.2. Study Population

We included 447 older patients between October 2018 and July 2019. Inclusion criteria: patients over 70 years old admitted to an acute geriatric or orthogeriatric unit with OD diagnosis during admission with the Volume-Viscosity Swallowing Test (V-VST) [12]. Exclusion criteria: end-of life situations, permanent low level of consciousness that precluded performance of the V-VST, enteral nutrition administered by nasogastric tube or percutaneous endoscopic gastrostomy, and refusal to participate. The study population inclusion procedure has also been published elsewhere [2,19].

All patients were informed about the study and gave their written consent. The study protocol was approved by the ethics review board of Hospital Universitario Ramón y Cajal (protocol Number 120/18) and was conducted following the principles and rules of the Declaration of Helsinki.

### 2.3. Health Status, Comorbidity and Geriatric Assessment, and In-Hospital Evolution

During hospital admission, a trained nurse collected demographic information, previous comorbidities and pharmacological treatment related to OD, geriatric assessment measures, the reason for hospitalization, presence of delirium during hospitalization, length of stay, and place of discharge, previously described in detail [2,19].

Regarding geriatric assessment, the nurse collected functional status with Barthel index of basic activities of daily living (ADL), Lawton index of instrumental ADL and Functional Ambulation Classification of gait; cognitive status using Global Deterioration Scale; and nutritional status through Mini Nutritional Assessment-Short Form and Body Mass Index, and calculated the body mass index. 

### 2.4. OD Assessment

All patients were clinically assessed for OD by a trained nurse by a general exam and the V-VST, within 48 h of admission in the acute geriatric unit and 72 h post-surgery in hip fracture patients. This evaluation was performed in the patients´ room and in the presence of the family or caregiver. If the patient presented low level of consciousness, the assessment was delayed, and in case of no improvement of the level of consciousness, the patient was excluded, as is described in Figure 1. Previous to the study, the nurse received theoretical and practical training about clinical diagnosis and management of OD in older persons by a Speech-Language Pathologist. 

The VVS-T uses different volumes and viscosities for evaluating safety signs of swallowing (cough, decreased oxygen saturation, and voice changes) and efficacy signs (poor labial seal, multiple swallows, and pharyngeal residue). This test is not only a clinical tool for screening, but also for diagnosis of OD with high sensitivity (93.2%) and a specificity (81.4%) for clinical diagnosis of OD, including severity, and it is simple and fast to perform [1,20]. This tool can be used to provide accurate indications on the optimal bolus volume and viscosity for dysphagia patients [1], as it was used in the recent study of older patients with dementia and dysphagia [18].

### 2.5. Dysphagia Management, Educational Intervention and In-Hospital Adherence Assessment

When OD was detected by the V-VST, the nurse orally informed of the diagnosis and the protocol design. She also explained the treatment recommendations, as well as the reason for these measures, both to the patient and to the caregiver and gave written specific, individualized recommendations about food texture (regular diet, soft diet, pureed diet, or dysphagia diet), liquid texture (water, nectar or pudding), liquid volume (5, 10 and or 20 mL), posture (sitting, chin tuck, head rotation) and delivery method advised (spoon or dysphagia cup). 

In-hospital adherence to these five recommendations was evaluated two days after diagnosis during mealtime by the same nurse´s direct observation in a dichotomous way (yes or no). This evaluation was performed after explaining the study, being the patient and the caregiver aware of the observation, as is presented in Figure 1. 

### 2.6. One-Month Self-Reported Adherence and OD-Related Complications Assessment

The nurse recorded the self-reported adherence to the measures recommended during hospital admission on a 3-point scale (always, sometimes, and never) by a telephone interview with patients or caregivers one month after hospital discharge, as summarized in Figure 1. She collected in the same call any reported complications, including respiratory infection, use of antibiotics, weight loss (≥3 kg), transfers to the emergency department, hospitalization, and mortality. Reported adherence and complications was also assessed with the main caregiver in those patients who had died one month after discharge

### 2.7. Data Analysis

Statistical analysis was performed using SPSS version 20. Data were described by relative and absolute frequencies or mean ± standard deviation as appropriate, and compared with the chi-square test, the Fisher exact test, the Mann–Whitney U test, and the Student’s *t*-test. 

In-hospital and one-month adherence rates were calculated for the five treatments: modified diet, texture and volume of liquid, feeding posture, and delivery method. As it was described previously, in-hospital adherence was assessed in a dichotomous was, while one-month adhere on a 3-point scale that was codified in two categories as it was performed by Low [21].

A patient was considered compliant when he/she was following the suggested recommendations, including those that should continue using a regular diet, water, or no delivery methods). However, compliance rates for individual treatments are reported only for patients with changes in these indications in order to avoid overestimating of these rates. We also used a univariate and multivariate regression model to analyze one-month complications risk factors. Statistical significance was set at *p* < 0.05.

## 3. Results

### 3.1. Sample Characteristics 

A total of 447 patients presented OD (97.8% with low efficacy signs and 64% with safety signs) and accepted participation in the study. Among included patients, 271 participants were admitted to the acute geriatric unit and 176 to the orthogeriatric unit, and 403 were followed after one month. The mean age was 92 years, 70.7% were women, and 25.5% lived in a nursing home before admission. Most had impaired functional and mental status, malnutrition, a high number of comorbidities, and polypharmacy. Of the patients, 60.6% had been admitted to the acute geriatric unit, mainly for a respiratory infection in 32.2%. Baseline information is reported in Table 1. 

### 3.2. OD Management Recommendations and Post-Discharge Complications

**Figure 2:**
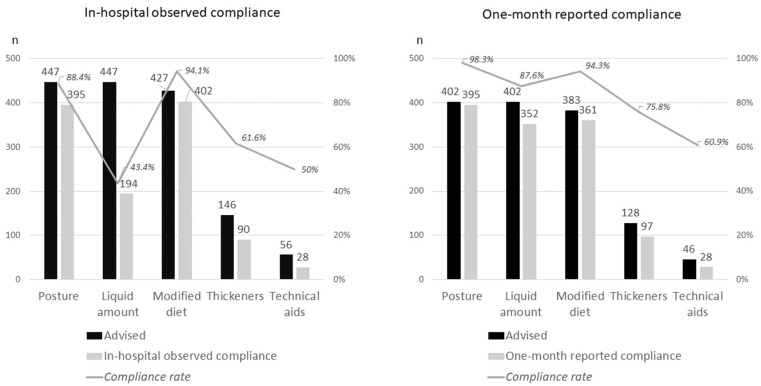
Management recommendations compliance rates during hospitalization and at one month of discharge.

The most frequent management recommendations were sitting when feeding and volume of liquid, which were recommended to all patients (*n* = 447), followed by the use of modified texture food (95.5% *n* = 427), self-feeding (56.8% *n* = 254), use of fluid thickeners (32.7% *n* = 146), and delivery method (12.5% *n* = 56), as is shown in Figure 2.

During hospitalization, 60.2% of patients presented delirium, and the mean length of stay was eight days for the overall sample. At discharge, 19.9% received oral nutritional supplements (ONS), and 31.5% were transferred to a nursing home, as is reported in Table 1.

Among patients assessed one month after discharge, 17.6% had been transferred to the emergency department or readmitted to the hospital, 15.6% reported weight loss, 8.9% a chest infection, and 6% had died. One-month complications are described in Table 1. 

### 3.3. Observed Adherence to Recommendations during Hospitalization

The following compliance rates were observed at the hospital follow-up visit: 88.4% for sitting feeding (395/447), 43.3% for liquid volume (194/447), 94.1% for food texture (402/427), 61.6% for fluid thickener (90/146), and 50% for technical aid (28/56), as shown in Figure 1.

Around one third (37.1%) of the participants followed all recommendations measures during hospitalization (166/447, Table 1). Non-compliance was more common in older patients (92 vs. 91 years old, *p* = 0.003) and in those previously living in a nursing home (28.9% vs. 19.9%, *p* = 0.036). Non-compliant patients had a worse functional status (Barthel index < 40 in 42.6% vs. 17.9%, *p* < 0.001), cognitive status (GDS ≥ 4 63.0% vs. 39.3%, *p* < 0.001), and nutritional status (MNA-SF ≤ 7 in 39.3% vs. 17.1%, *p* < 0.001). 

Non-compliant patients had a significantly higher prevalence of dementia (33.1% vs. 17.5%, *p* < 0.001) and stroke (16.4% vs. 9.0%, *p* = 0.029). Admission in the acute geriatric unit vs. the orthogeriatric ward was also associated with non-adherence (68.3% vs. 47.6%, *p* < 0.001). Respiratory infection, one of the main OD complications, as the main reason for admission was related to reduced adherence (35.9% vs. 25.9%, *p* = 0.028). Non-compliance was higher in patients with dysphagia safety signs (75.4% vs. 44.6%, <0.001), while there was a higher in-hospital adherence in patients advised to self-feed (80.7% vs. 42.7%, *p* < 0.001). However, there were no differences in adherence to OD measures according to the presence of efficacy signs, delirium during hospitalization, or one-month complications.

### 3.4. One-Month Self-Reported Adherence to Recommendations

Most patients (76.4%) reported following all recommendations during the month after hospitalization (308/403), as is presented in Table 2. One-month self-reported compliance rates were: 98.3% for sitting feeding (395/402), 87.6% for the liquid volume (352/402), 94.3% for food texture (361/383), 75.8% for fluid thickener (97/128), and 60.9% for technical aid (28/46), as shown in Figure 1. Similar adherence rates were observed during hospitalization and at one month, with higher follow-up rates for sitting feeding and for food texture, and a significant increase in adherence rate after discharge regarding the liquid volume (43.3% during admission and 87.5% after discharge) and the use of thickeners (61.6% vs. 75.8%). 

One-month reported compliance to OD measures was more common in patients living in a nursing home (33.4% vs. 20%, *p* = 0.013) and when a professional caregiver (as opposed to an informal caregiver) was in charge (28.6% vs. 14.9%, *p* = 0.008). In addition, non-compliant patients had a worse cognitive status (GDS ≥ 4 65.8% vs. 51.2%, *p* < 0.024) and nutritional status (MNA-SF ≤ 7 in 45.1% vs. 27.3%, *p* < 0.001). Non-compliance was higher in patients with safety signs of OD (76.8% vs. 58.4%, *p* = 0.001). Moreover, one-month compliance was associated with in-hospital adherence (43.2% vs. 28.4%, *p* = 0.01). Non-compliant patients at one month presented more commonly weight loss (24.2% vs. 13.0%, *p* = 0.008) and had higher mortality rates (16.8% vs. 2.6%, *p* < 0.001).

### 3.5. OD Adverse Outcomes

In univariate analysis, mortality at one month after hospital discharge was higher in older patients (95 vs. 92 years, *p* = 0.029), and in those with functional impairment measured by a Barthel index <40 (69.6% vs. 30.6%, *p* > 0.001), a body mass index (BMI) < 22 (41.7% vs. 16.3%, *p* = 0.004), and delirium during hospitalization (79.2% vs. 58.6%, *p* = 0.046). One-month mortality was also associated with self-reported adherence rates: global adherence (33% vs. 79.2%, *p* < 0.001), diet (75% vs. 95.8%, *p* < 0.001), liquid volume (45.8% vs. 90.2%, *p* < 0.001), and sitting feeding (79.2% vs. 99.5%, *p* < 0.001), as is presented in Table 3.

Other OD complications (respiratory infection, use of antibiotics, weight loss, transfers to the emergency department and hospitalization), at one month after discharge were also associated with gender (65% vs. 75.4%, *p* = 0.033), Barthel index <40 (40% vs. 29.8%, *p* = 0.048) and BMI < 22 (27% vs. 13.8%, *p* = 0.001). OD complications were also more frequent in non-compliant patients (81.1% vs. 65.9%, *p* = 0.001), to diet (97.5% vs. 87.8%, *p* < 0.001), to adaptation of the texture (94.6% vs. 87%, *p* = 0.008), and to liquid volume (90.7% vs. 80.5%, *p* = 0.004).

On multivariable regression analysis, summarized in Table 4, the strongest risk factor associated with one-month mortality was BMI < 22 (OR 4.65, *p* = 0.003, 95% CI 1.66–13.02), followed by Barthel index < 40 (OR 4.37, *p* = 0.003, 95% CI 1.64–11.65) and one-month reported global adherence to recommendations as a protective factor (OR 0.12, *p* < 0.001, 95% CI 0.04–0.315). 

In addition, the strongest risk factor for other OD complications one month after discharge was a BMI < 22 (OR 2.51, *p* = 0.001, 95% CI 1.44–4.37), while one-month self-reported global adherence (OR 0.49, *p* = 0.005, 95% CI 0.30–0.81) and female gender (OR 0.52, *p* = 0.010, 95% CI 0.32–0.85) were protective.

## 4. Discussion

This study found a low observed compliance with individualized recommendations to manage dysphagia in very old hospitalized patients with OD (37.1%) with improved self-reported compliance in the first month after hospital discharge (76.4%). We are not aware of other studies that report both compliance rates. We found a higher adherence than that described in the systematic review of Krekeler (21.9–51.9) [17]. However, studies with similar methods found similar adherence rates during hospitalization (35.6–56.5%) [22,23,24] and after-hospitalization (>50–79%) [21,25,26]. The difference between in-hospital and after discharge compliance might be explained by the assessment method used: direct observation [22,23,24] vs. self-reported [18,21] or not described [25,26]. The low compliance rates found in our patients is worrying, especially in older patients admitted for a respiratory infection, one of the main complications of OD in older patients. Recently, the Mataró research group developed a modified Mediterranean diet to increase patient compliance [27].

Compliance with recommendations varied with the content: they were higher for feeding posture and food texture, and low for liquid volume and use of thickeners. Rosenvigne also found higher compliance during hospital assessments for dietary modifications (82% and 78%) than for liquid volume (35.3% and 69%) or consistency of fluids (48% and 64%) [22]. Shim also found low thickener compliance (56.5%) [23], while Espinosa Val found lower compliance to fluid adaptation (50.4%) than to diet adaptation (88.3%) [18]. In comparison, Low registered high reported compliance rates to consistency of diet (79%) and liquid (84%) [21], similar to our one-month self-report compliance rates of modified diet and liquid (94.1% and 75.8%). The lower adherence rates to the adaptation of fluid (quantity and texture), described in our study may be caused by the previously reported dissatisfaction of thickeners [13]. Still, there is also a high difference in observed and self-reported adherence rates to liquid volume in our study (43.3% vs. 87.6%), without any apparent reason except for the assessment method.

We included older patients admitted to hospital with OD due to different causes like other studies [18,21,22,23,24,25], but with essential differences in the methodology. Most of the previous studies were retrospective or case series [21,22,23,24] and included OD patients diagnosed by videofluoroscopy [21,23,24], while we performed a prospective study after diagnosis of OD with a validated and accessible clinical assessment, the V-VST [18,20].

We found lower adherence rates in patients with dementia (GDS ≥ 4), malnutrition (MNA-SF ≤ 7), and safety signs of OD. This association has not been reported previously. Patients with moderate to severe dementia depend on their caregivers and may not change their feeding habits due to their cognitive deficits [24]. This may be partially improved with professional caregivers. The association with malnutrition may be a consequence of undetected OD. The association between low adherence and safety signs is unexpected, as patients with safety concerns would be expected to have a drive to improve compliance. This might be explained due to the complexity of management recommendations for patients with impaired safety and more frequent recommendations in patients with OD safety signs of modified diets (97.6% vs. 91.9%, *p* = 0.006), fluid thickeners (49.7% vs. 2.5%, *p* < 0.001) and delivery method (15.7% vs. 6.8%, *p* = 0.006).

There was also an inverse association between compliance rates during and after hospitalization and living in a nursing home, which may be explained by the different assessment methods (observed vs. self-reported) and the presence of family caregivers of nursing home patients during hospital admission. We also found an association between in-hospital adherence with Barthel index, own feeding advice and age. Previously Low reported that adherence to OD management is associated with age and living at home [21], but Shim did not find any risk factor of measures compliance [23].

To our best knowledge, this is the first study that has shown a relationship between compliance one month after discharge, rated on a 3-point scale, and in-hospital adherence, rated dichotomously (43.2% vs. 28.4%, *p* = 0.010). Recently, Espinosa-Val observed that non-compliance patients during follow up reported non-compliance during admission, without adherence assessment during hospitalization [18]. This fact may allow focusing on training management during hospital admission in patients with an expected low compliance.

We found a lower one-month mortality in patients with good adherence to treatment recommendations in a multivariate analysis and higher risk among patients with low BMI or Barthel index. This is reassuring as compliance had not been included in previous mortality studies in patients with OD [5,6,25,26], except the study performed by Espinosa-Val, which did not observe an association between therapeutic compliance after hospitalization and mortality or other complications [18]. Moreover, 31% of our patients presented other complications in the month after hospital discharge. Multivariate analysis showed that risk was higher in patients with a low BMI and lower in women and in those with self-reported adherence to recommendations. Lower loss of weight in adherent patients to our recommendations could be justified by better oral intakes following hospital indications. Low previously found a lower risk or hospital admissions for chest infections in adherent patients [21], and recently Martín reported lower readmissions in patients with a minimal-massive intervention based on diet adaptation and oral hygiene [25].

Therefore, assessment and implementation of recommendations to treat OD may help in reducing complications after a hospital admission where OD is detected. However, a systematic review published in 2018 [13] proposed a weak recommendation against the use of texture-modified liquid according to the results of two trials [18,20]. The first study did not find differences in the incidence of pneumonia with the use of thickened liquid or chin down posture [26]. In contrast, the second study described a lower risk of aspiration with honey-thickened liquid [28]. While this review supported using texture-modified foods in patients with OD as a GCP [13], based on the IDSSI Framework that developed an international terminology for texture-modified foods and thickened fluids for OD patients [29].

Another Cochrane review [30] evaluated the same topic in patients with dysphagia and dementia, including the same trials [26,28], that were part of a large multicenter trial based on videofluoroscopy, with a high risk of bias, concluding the need for further high-quality trials. While the systematic review performed by Wu et al. in 2021, also remembered the low quality of evidence and the need for high-quality research [31] and recently, different authors started a debate about the use of modified diets highlighting the importance of shared decision-making [32] and patient compliance [33].

In our study, staff investment was low. A single nurse was trained to perform V-VST, explain management, and collect data. Previous studies usually involved speech and language therapists in the diagnosis of OD [21,22,24], but nurses have shown to effectively diagnose OD [2,5,6,19]. Also, a current systematic review described that 49% of the articles include nurses among the participants, mainly in screening and performing a clinical assessment of OD [34]. According to our study, the nurse can be a key member in managing OD, performing diagnosis, indicating individualized recommendations and training patients and caregivers.

Our study has some strengths: it is a prospective study, includes a high number of patients, and measures a range of demographic, clinical, and functional variables. We also used a validated assessment of dysphagia and two different methods to assess adherence (observation vs self-reported) that have to be considered in the compliance rate comparison. We recorded hospital adherence based on one-time direct observation and one-month adherence self-reported. The main limitation of the study is the subjectivity of this self-reported adherence to measures, especially in patients who died during follow-up. However, most studies described in the systematic review assessed compliance through journals, checklists or diaries [17]. We also performed a single telephone follow-up, fewer than other similar studies [5,6,25,26], limiting the multivariate analysis due to the low number of one-month complications, which precluded individual analysis of each complication. We also based OD diagnosis in V-VST, with a lack of instrumental assessment due to the complex access of this acute population [35] and based recommendations on clinical assessment that was performed by one trained nurse that also evaluated the adherence during and after hospitalization. Despite our efforts on the educational intervention to patients and caregivers about OD therapeutic adherence, assessing adherence to each recommendation during hospitalization and after one month, there is still room for improvement when adapting OD recommendations to each patient with good compliance. Moreover, more studies are needed to study the association between adherence to treatment and OD outcomes.

## 5. Conclusions

Compliance with treatment recommendations for OD in older hospitalized patients was low and increased after hospital discharge. One-month self-reported adherence to recommendations was associated with lower rates of mortality and other complications associated with OD. More research is needed to address how to improve compliance with recommendations and to explore if a higher adherence is associated with better outcomes.

## Figures and Tables

**Table 1:** Baseline characteristics of patients and factors related to in-hospital adherence ^a^ to recommendations.

Variable	Characteristic	Total *n* = 447	Adherence during Hospitalization *n* = 166	Non Adherence during Hospitalization *n* = 281	*p* Value
Demographic	Age (years) ^c^	92 (88; 95)	91 (86; 95)	92 (89; 96)	**0.003**
	Female ^b^	316 (70.7%)	116 (69.9%)	200 (71.2%)	0.771
	Living in nursing home ^b^	114 (25.5%)	33 (19.9%)	81 (28.9%)	**0.036**
Geriatric Assessment	Barthel Index < 40 ^b^	147 (33.5%)	29 (17.9%)	118 (42.6%)	**<0.001**
	Lawton 0 ^b^	244 (57.2%)	68 (42.5%)	177 (66.0%)	**<0.001**
	FAC ≤ 3 ^b^	244 (57.1%)	69 (43.4%)	175 (65.3%)	**<0.001**
	GDS ≥ 4 ^b^	203 (54.4%)	53 (39.3%)	150 (63.0%)	**<0.001**
	MNA-SF ≤ 7 ^b^	122 (31%)	25 (15.1%)	97 (39.3%)	**<0.001**
	BMI < 22 ^b^	80 (18.1%)	29 (17.5%)	51 (18.9%)	0.848
	Professional caregiver ^b^	60 (13.4%)	22 (13.3%)	38 (13.5%)	0.681
Comorbidities and previous treatment	Dementia ^b^	122 (27.3%)	29 (17.5%)	93 (33.1%)	**<0.001**
	Vascular disease ^b^	119 (26.6%)	37 (22.3%)	82 (29.2%)	0.111
	Stroke ^b^	61 (13.6%)	15 (9.0%)	46 (16.4%)	**0.029**
	Parkinson ^b^	30 (6.7%)	12 (7.2%)	18 (6.4%)	0.737
	Head and neck cancer ^b^	18 (4.0%)	4 (2.4%)	14 (5.0%)	0.181
	Malnutrition ^b^	10 (2.2%)	2 (1.2%)	8 (2.8%)	0.335
	Number of drugs ^c^	8 (6; 10)	8 (5; 10)	8 (6; 8)	0.149
Unit of admission	Acute Geriatric Unit ^b^	271 (60.6%)	79 (47.6%)	192 (68.3%)	**<0.001**
Main reason for admission	Respiratory infection ^b^	144 (32.2%)	43 (25.9%)	101 (35.9%)	**0.028**
Dysphagia assessment	Safety sign ^b^	286 (64.0%)	74 (44.6%)	212 (75.4%)	**<0.001**
	Efficacy sign ^b^	437 (97.8%)	161 (97.0%)	276 (98.2%)	0.510
	Self feeding advised ^b^	254 (56.8%)	134 (80.7%)	120 (42.7%)	**<0.001**
	Professional caregiver present ^b^	20 (4.6%)	9 (5.7%)	11 (4.0%)	0.437
In-hospital complications and discharge	Delirium ^b^	269 (60.2%)	92 (55.4%)	177 (63.0%)	0.114
	Length of stay (days) ^c^	8 (5; 12)	8 (5; 13)	8 (5; 12)	0.405
	ONS at discharge ^b^	80 (19.9%)	38 (23.8%)	42 (17.4%)	0.116
	Nursing home ^b^	131 (31.8%)	42 (28.4%)	89 (33.7%)	0.265
1 month complications	Chest infection or antibiotic use ^b^	36 (8.9%)	12 (7.5%)	24 (9.9%)	0.413
	Weight loss ≥ 3 Kg ^b^	63 (15.6%)	28 (17.5%)	35 (14.4%)	0.402
	Emergency department referral or hospital admission ^b^	71 (17.6%)	22 (13.8%)	49 (20.2%)	0.098
	Death ^b^	24 (6%)	7 (4.4%)	17 (7.0%)	0.277

Abbreviations: FAC: Functional Ambulation Classification, GDS: Global Deterioration Scale; MNA-SF Mini Nutritional Assessment Short Form; BMI: Body Mass Index; ONS: Oral Nutritional Supplements. Significant level is set at 5% and marked with bold font. ^a^ Adherence during hospitalization to five measures indicated. ^b^ *n* (%), Pearson Chi-Square test. ^c^ Median (Q1; Q3), Mann–Whitney U test.

**Table 2:** Clinical data according to one-month self-report adherence.

Variables		Always/Sometimes Complied with Advice *n* = 308	Never Complied with Advice *n* = 95	*p* Value
Demographic	Age ^b^	92 (88; 95)	92 (88; 95)	0.996
	Female ^a^	228 (74.0%)	63 (66.3%)	0.142
	Discharge to nursing home ^a^	103 (33.4%)	19 (20.0%)	**0.013**
	Professional caregiver interviewed ^a^	86 (28.6%)	14 (14.9%)	**0.008**
Geriatric assessment	Barthel Index < 40 ^a^	92 (30.4%)	38 (41.3%)	0.050
	Lawton 0 ^a^	169 (57.5%)	52 (57.8%)	0.961
	FAC ≤ 3 ^a^	167 (56.6%)	51 (57.3%)	0.908
	GDS ≥ 4 ^a^	133 (51.2%)	50 (65.8%)	**0.024**
	MNA-SF ≤ 7 ^a^	74 (27.3%)	36 (45.0%)	**0.003**
	BMI < 22 ^a^	49 (16.1%)	22 (23.4%)	0.107
Comorbidities and previous treatment	Dementia ^a^	81 (26.3%)	31 (32.6%)	0.228
	Vascular disease ^a^	77 (25.0%)	29 (30.5%)	0.285
	Stroke ^a^	44 (14.3%)	10 (10.5%)	0.347
	Parkinson ^a^	23 (7.5%)	5 (5.3%)	0.460
	Head and neck cancer ^a^	12 (3.9%)	5 (5.3%)	0.563
	Malnutrition ^a^	5 (1.6%)	3 (3.2%)	0.399
	Number of drugs ^b^	8 (6; 10)	8 (6; 10)	0.826
Current hospitalization	Acute Geriatric Unit ^a^	181 (58.5%)	64 (67.4%)	0.133
	Chest infection ^b^	98 (31.8%)	31 (32.6%)	0.882
	Delirium ^a^	178 (57.7%)	63 (66.3%)	0.139
	Length of stay ^b^	7 (5; 12)	8 (5; 12)	0.807
Dysphagia signs and indications during hospitalization	Safety sign ^a^	180 (58.4%)	73 (76.8%)	**0.001**
	Efficacy sign ^a^	303 (98.4%)	90 (94.7%)	0.060
	Written indications ^a^	253 (82.1%)	80 (84.2%)	0.642
	OD diagnosis in medical report ^a^	116 (37.7%)	44 (46.3%)	0.132
	OD diagnosis in nurse report ^a^	24 (7.8%)	7 (7.4%)	0.892
	In-hospital observed adherence ^a^	133 (43.2%)	23 (28.4%)	**0.010**
One-month complications	Chest infection or use of antibiotics ^a^	25 (8.1%)	11 (11.6%)	0.301
	Weight loss ≥ 3 kg ^a^	40 (13.0%)	23 (24.2%)	**0.008**
	Emergency department referal or hospital admission ^a^	49 (15.9%)	22 (23.2%)	0.105
	Death ^a^	8 (2.6%)	16 (16.8%)	**<0.001**

Abbreviations: FAC: Functional Ambulation Classification, GDS: Global Deterioration Scale; MNA-SF: Mini Nutritional Assessment Short Form; BMI: Body Mass Index. Significant level is set at 5% and marked with bold font. ^a^ *n* (%), Pearson Chi-Square test. ^b^ Median (Q1; Q3), Mann-Whitney U test.

**Table 3:** Demographic characteristics and clinical data according to mortality or one-month complications in the univariate analysis.

Variables		Death			Other OD Complications		
		Yes *n* = 24	No *n* = 379	*p* Value	Yes *n* = 123	No *n* = 280	*p* Value
Demographic	Age ^a^	95 (89; 98)	92 (88; 95)	**0.029**	92 (89; 95)	92 (87; 95)	0.450
	Female ^b^	62.5	72.8	0.274	65.0	75.4	**0.033**
	Discharge to nursing home	25.6	30.6	0.562	27.6	31.4	0.446
Geriatric assessment	Barthel Index < 40 ^b^	69.6	30.6	**<0.001**	40.0	29.8	**0.048**
	Lawton 0 ^b^	78.3	56.2	**0.038**	61.7	55.8	0.278
	FAC ≤ 3 ^b^	78.3	55.4	**0.032**	67.5	52.1	**0.005**
	GDS ≥ 4 ^b^	60.9	54.1	0.608	57.0	53.4	0.543
	MNA-SF ≤ 7 ^b^	47.8	30.2	0.078	42.1	26.6	**0.004**
	BMI < 22 ^b^	41.7	16.3	**0.004**	27.0	13.8	**0.001**
Comorbidities	Dementia ^b^	25.0	28.0	0.753	28.5	27.5	0.844
	Delirium during hospitalization	79.2	58.6	**0.046**	64.2	57.9	0.230
	Vascular disease ^b^	16.7	26.9	0.269	27.6	25.7	0.686
	Stroke ^b^	20.8	12.9	0.347	14.6	12.9	0.630
	Parkinson ^b^	8.3	6.9	0.679	8.9	6.1	0.296
	Number of drugs ^a^	8 (6;11)	8 (5; 10)	0.509	8 (6; 11)	8 (5; 10)	0.239
Dysphagia assessment	Safety sign ^b^	70.8	62.3	0.400	68.3	60.4	0.129
	Efficacy sign ^b^	95.8	97.6	0.463	95.9	98.2	0.181
Intrahospital adherence (observed)	Global adherence ^b^	29.2	40.4	0.277	38.2	40.4	0.685
	Adherence to diet ^b^	87.5	95.0	0.135	96.7	93.6	0.196
	Adherence to textures of liquids ^b^	91.	88.4	1.000	86.2	89.6	0.314
	Adherence to liquid volumes ^b^	41.7	46.7	0.631	44.7	47.1	0.653
	Adhence to posture ^b^	75.0	88.4	0.100	85.4	88.6	0.369
	Adherence to technical aids ^b^	83.3	93.1	0.093	91.1	93.2	0.447
One-month reported adherence (reported)	Global adherence ^b^	33.3	79.2	**<0.001**	65.9	81.1	**0.001**
	Adherence to diet ^b^	75.0	95.8	**0.001**	87.8	97.5	**<0.001**
	Adherence to textures of liquids ^b^	83.3	92.9	0.103	87.0	94.6	**0.008**
	Adherence to liquid volumes ^b^	45.8	90.2	**<0.001**	80.5	90.7	**0.004**
	Adherence to posture ^b^	79.2	99.5	**<0.001**	96.7	98.9	0.208
	Adherence to delivery methods ^b^	87.5	96.0	0.084	96.7	95.0	0.430

Abbreviations: FAC: Functional Ambulation Classification, GDS: Global Deterioration Scale; MNA-SF Mini Nutritional Assessment Short Form; BMI: Body Mass Index. Significant level is set at 5% and marked with bold font. ^a^ Median (Q1; Q3), Mann-Whitney U test. ^b^ %, Pearson Chi-Square test.

**Table 4:** Multivariate Cox Analysis: Variables associated with death and other complications associated with dysphagia one month after hospitalization.

Model	Variable	OR	95% CI	*p* Value
Number 1: Mortality	Age (per year)	1.09	1.00–1.19	0.051
	Female	0.47	0.17–1.30	0.146
	Barthel Index < 40	4.37	1.64–11.65	**0.003**
	Body Mass Index < 22	4.65	1.66–13.02	**0.003**
	One-month self reported global adherence	0.12	0.04–0.315	**<0.001**
Model 2: Other complications associated with dysphagia	Age (per year)	1.00	0.97–1.04	0.811
	Female	0.52	0.32–0.85	**0.010**
	Barthel Index < 40	1.50	0.93–2.41	0.094
	Body Mass Index < 22	2.51	1.44–4.37	**0.001**
	One-month self reported global adherence	0.49	0.30–0.81	**0.005**

Significant level is set at 5% and marked with bold font.

## Data Availability

The data presented in this study are available on request from the corresponding author. The data are not publicly available due to privacy and permission restricted to the publication of this article only.
